# The Effects of Caffeine on Sleep and Maturational Markers in the Rat

**DOI:** 10.1371/journal.pone.0072539

**Published:** 2013-09-04

**Authors:** Nadja Olini, Salomé Kurth, Reto Huber

**Affiliations:** 1 Child Development Center, University Children's Hospital Zurich, Zurich, Switzerland; 2 Neuroscience Center Zurich (ZNZ), University and ETH Zurich, Zurich, Switzerland; 3 University of Colorado Boulder, Department of Integrative Physiology, Boulder, Colorado, United States of America; 4 Zurich Center for Integrative Human Physiology (ZIHP), University of Zurich, Zurich, Switzerland; Imperial College London, United Kingdom

## Abstract

Adolescence is a critical period for brain maturation during which a massive reorganization of cortical connectivity takes place. In humans, slow wave activity (<4.5 Hz) during NREM sleep was proposed to reflect cortical maturation which relies on use-dependent processes. A stimulant like caffeine, whose consumption has recently increased especially in adolescents, is known to affect sleep wake regulation.

The goal of this study was to establish a rat model allowing to assess the relationship between cortical maturation and sleep and to further investigate how these parameters are affected by caffeine consumption. To do so, we assessed sleep and markers of maturation by electrophysiological recordings, behavioral and structural readouts in the juvenile rat. Our results show that sleep slow wave activity follows a similar inverted U-shape trajectory as already known in humans. Caffeine treatment exerted short-term stimulating effects and altered the trajectory of slow wave activity. Moreover, caffeine affected behavioral and structural markers of maturation. Thus, caffeine consumption during a critical developmental period shows long lasting effects on sleep and brain maturation.

## Introduction

Caffeine is the most widely consumed stimulant. It is known that caffeine consumption, in particular among adolescents, has increased significantly in recent years [1]. The major effect of moderate caffeine consumption in the central nervous system is to block adenosine A_1_ and A_2A_ receptors [2], which are present in almost all brain areas [3], [4]. As a stimulant, caffeine has clear wake-promoting effects [5]. Several studies have shown that caffeine diminishes the build up of sleep pressure during wakefulness [2], [6], [7]. Thus, after caffeine application, the major electrophysiological marker of sleep pressure, EEG slow wave activity (SWA, <4.5 Hz) during non-rapid eye movement (NREM) sleep was reduced [8]. In contrast, adenosine agonists seem to promote sleep, in particular NREM sleep [6], [9], [10]. Together these studies show that caffeine has significant impacts on sleep wake regulation.

Adolescence is a critical period for brain development which is characterized by extensive morphological and functional changes [11]. In humans it was shown that maximal cortical synapse density is reached shortly before puberty, followed by a reduction in synapse density during adolescence [12]. These massive changes in connectivity are based on an overproduction of synapses during early development [13], which is followed by a net elimination of synapses [14]. Such processes of cortical reorganization have been linked to activity-dependent processes [15]. Studies show that behavior-dependent neuronal activity is needed for cortical maturation [16], [17].

Interestingly, the inverted U-shaped trajectory of synapse density is paralleled by changes in SWA [18], [19], [20]. In humans, SWA increases in pre-pubertal children, reaches a maximum around puberty and decreases during adolescence. A convincing explanation for the relationship between SWA and synapse density comes from our knowledge about how slow waves are generated. SWA during NREM sleep is characterized at the cellular level by a specific neuronal firing pattern consisting of an alternation between a depolarized up state, when neurons keep firing, and a hyperpolarized down state, when neurons are silent. Intracellular recordings have shown that during deep NREM sleep virtually every cortical neuron is included in such slow oscillations [21], [22]. Furthermore, a study by Vyazovskiy et al. [23] discovered by means of multi-unit recordings in the rat a close relationship between sleep pressure and the synchronization of cortical neuronal activity during sleep. More specifically, the wakefulness-related increase in the amplitude of slow waves, as reflected by more SWA on the surface EEG, was associated with a more synchronous firing pattern. In the course of subsequent sleep neuronal synchrony was reduced. Interestingly, measures of synaptic strength follow a similar time course, i.e. show a net increase during wakefulness and decrease during sleep [24], [25]. As numerous studies have shown is increased synaptic strength associated with an increased level of neuronal activity synchronization [26]. These observations may suggest that changes in synaptic connectivity affect the level of synchronization which relates to corresponding SWA changes. All together these studies support a close relationship between cortical plasticity and SWA. Since cortical maturation involves vast plasticity processes, which are use-dependent, the question arises whether the application of a stimulant during this critical period affects the relationship between sleep SWA and cortical plasticity. To address this question the aim of this study was 1) to establish a juvenile rat model allowing to investigate the relationship between brain maturation and sleep, 2) to assess the effects of caffeine on sleep in these juvenile rats, and 3) to test whether such a caffeine application affects markers of brain maturation.

We performed longitudinal electrocortical recordings (ECoG) in 28 male Sprague Dawley rats. In addition, we assessed behavioral and anatomical development by means of behavioral testing and immunohistochemistry, respectively. Our results show a similar trajectory of SWA in the rat as already found in humans. Moreover, caffeine intake during the period when under sham condition the sleep SWA trajectory started to decrease resulted in a delay of all three assessed markers of brain maturation.

## Methods

### Surgical procedures

Animal protocols followed the National Institutes of Health Guide for the Care and Use of Laboratory Animals and facilities and were approved by the Cantonal Veterinary Office of Zurich.

Animals were delivered after weaning with 22 days of age. To immediately acclimatize the animals to the recording box all animals were placed in their box the day they arrived, 3 days before the recording started. Then, surgery was performed in 23- to 25-day old male Sprague Dawley rats according to a protocol published previously [27]. Under isoflurane anesthesia all animals were implanted epidurally with gold-plated miniature screws (0.9 mm diameter) for electrocortical recording (ECoG) [right hemisphere: frontal, 1.5 mm anterior to bregma, 2 mm lateral to the midline and parietal, 2 mm anterior to lambda, 2 mm lateral to midline; reference: above cerebellum, 2 mm posterior to lambda, on the midline]. Two gold wires (0.2 mm diameter) were inserted bilaterally into the neck muscles for electromyogram (EMG) recording. The electrodes were connected to stainless-steel wires and fixed to the skull with dental acrylic cement. All animals received a single dose of postoperative analgesic Temgesic (0.1 mg/kg Buprenorphin, s.c.) during the last 30 min of the surgery. After the surgery no animal lost weight, instead all animals showed normal weight gain across the experiment. At the end of the experiment all brains were carefully inspected and no cranial damage was observed. Histological analyses at the location of the previously positioned screws did not show any abnormalities.

### Electrocortical recordings

For longitudinal recording the rats were connected by a fine cable to a swivel and remained connected throughout the experiment. Data collection started immediately after surgery for 20 consecutive days. Animals were singly housed and kept under a 12-hour light (9 am to 9 pm) and 12-hour dark period. Food and water were given ad libitum. The ECoG and EMG signals were amplified (amplification factor, ∼2000), filtered (highpass filter: –3 dB at 0.016 Hz; low-pass filter: –3 dB at 40 Hz) sampled with 512 Hz, digitally filtered [ECoG: low-pass finite impulse response (FIR) filter, 25 Hz; EMG: bandpass FIR filter, 20–50 Hz], and stored with a resolution of 128 Hz. The ECoG power spectra were computed for 4s epochs by a fast Fourier transform routine. Adjacent 0.25 Hz bins were averaged into 0.5 Hz (0.25–5 Hz) and 1.0 Hz (5.25–25 Hz) bins. Before each recording, the EEG and EMG channels were calibrated with a 10 Hz, 300 μV peak-to-peak sine wave.

Vigilance states (NREM, REM and wake) were visually determined, as previously documented [27], by off-line visual inspection of the ECoG and EMG signals. Epochs containing artifacts in one derivation were excluded from spectral analysis of both ECoG derivations. As a representative time window the first 3 hours after light onset were used to determine vigilance states for every day. In addition, vigilance states for the entire 24 hours were determined for selected days throughout the longitudinal recordings: postnatal day 29 (P29)-P31, P35 and P38. Vigilance states could always be determined. Sample EEG traces are presented to indicate age and condition-dependent changes. To do so, the 4-s epoch scored as NREM sleep that exhibited highest SWA at the beginning of the light period was chosen for a representative sham and caffeine treated animal. Data analyses and statistics were performed using the MATLAB software package (MathWorks). Contrasts were tested by post-hoc parametric or non-parametric statistical tests after significance by ANOVA.

### Caffeine application

We performed a pilot experiment to assess caffeine consumption and its dose-dependent effect on locomotor activity. We were aiming for a caffeine dosage which would minimally affect the amount of wakefulness but rather affect SWA during a specific time interval. Thus, in the pilot experiment we administered 3 different caffeine dosages via the drinking water (0.00 g/l, 0.15 g/l and 0.3 g/l). As in the main experiment, in this pilot experiment caffeine was administered between P30 and P34 during 8 hours per day from 6 pm to 2 am. This time window contained the last 3 hours of the light period and the first 5 hours of the dark period. We chose this time window since we aimed at having maximal caffeine concentration at the beginning of the dark period when the animals are naturally awake and leave enough time in the second half of the dark period for a wash out. With this approach we were hoping 1) to induce as little additional wakefulness as possible and 2) with a half life time ∼1 h [2] to have as little caffeine in the circulation as possible once the main sleep period starts (at the beginning of the light period). We performed actimetry but no ECoG in these pilot animals. All water bottles were quickly exchanged at these two time points and water and caffeine consumption was calculated based on the weight reduction of the water bottles. The results of the pilot experiment showed that overall locomotor activity across 24 hours was increased on the first day of caffeine treatment between the low caffeine consumers (0.15 g/l) compared to sham treated animals. However, at this dosage, during the remaining 4 days of caffeine treatment no changes in general locomotor activity was found. Based on this result, the same protocol was applied in the main experiment using the lower caffeine dosage (0.15 g/l, n = 11 animals). In addition, based on the consistent and stable daily liquid intake across 8 and 16 hours found during our pilot experiment we expected similar caffeine consumption in our main experiment. Indeed, caffeine consumption did not differ between our pilot and the main experiment. In our main experiment water or caffeine consumption did not differ across 8 hours or 16 hours or across the entire 24 hours. The overall daily liquid intake was 24.5±1.6 ml/100 g body weight in sham treated animals and 24.8±1.6 ml/100 g body weight in caffeine treated animals. This is in line with a previous study showing a daily water intake of ∼26 ml/100 g body weight at the age of the 5^th^ postnatal week [28].

Maximal daily caffeine intake was calculated based on the rat's weight (at 6 pm) and resulted in a mean caffeine intake of 16.0±1.4 mg/kg per day. Because caffeine metabolism differs between rodents and humans, this dosage corresponds to ∼5 mg/kg in humans [2], which is about 3 to 4 cups of coffee (each containing ∼100 mg caffeine) [2]. The same procedure of changing water bottles twice a day and daily weighting was applied in sham treated animals (n = 17). Experiments of caffeine and sham treated animals were run in parallel.

### Behavioral tests

A free exploration task was performed in a subset of 8 sham and 9 caffeine treated rats at the ages P28 and P42 between 4.30 pm and 5.30 pm. During the test phase, each rat remained in its home cage and was exposed to a new object for 1 hour and its behavior was video recorded (Roline, RIC-45 IP Camera). The same procedure was repeated at an older age (P42) with a new object. The objects were counter-balanced among individuals. Offline, the predominant occurred behavior (quiet waking, grooming, exploring, object exploring) was determined for each 10s interval for 1 hour per rat. In the results the 4 different behaviors are expressed as a percentage of the sum of all intervals with a behavior.

### Immunohistochemistry

Based on evidence in non-human primates that the entire cerebral cortex matures as an integrated network rather than as a system-by-system cascade [29], we expected similar changes in the different neurotransmitter systems. Previous publications in the rat showed extensive maturational processes in the dopaminergic and in the cholinergic system during the preadolescent period [30], [31]. Moreover, there is good evidence suggesting a direct involvement of the cholinergic system in cortical development [32], [33], [34], [35]. For example, cholinergic innervation is needed for the correct maturation of cortical barrels, which relies on plasticity processes [35]. Thus, cholinergic terminals seem to be a representative marker for overall cortical development. However, the development of different neuromodulators may vary in time and space [36], [37] and, as a consequence, may show a deviation from overall cortical development. With this limitation in mind we used the changes in the cholinergic system across age as a marker of cortical maturation. Therefore, we performed immunohistochemistry using DAB immunoperoxidase reaction to assess the vesicular acetylcholine transporter (VAChT) protein, mostly present in presynaptic terminals. To do so, a subset of different animals was perfused at two time points P30 and P42 either after sham or caffeine treatment (n = 6 per group). The animals were deeply anesthetized with an overdose of isoflurane and perfused through the ascending aorta with PBS (pH 7.4), followed by 4% paraformaldehyde in PBS. Then the brain was removed from the skull and postfixed overnight in the same fixative solution.

Afterwards the brains were rinsed in PBS, cryoprotected in 30% sucrose solution in PBS and frozen at –80°C. Coronal sections were then cut from frozen blocks with a sliding microtome (40 μm) and stored at –20°C in anti-freeze. Further processing was performed according to a protocol published previously [38]. To label VAChT, presumably reflecting cholinergic presynaptic terminals, the sections were incubated overnight at 4°C in the primary antibody solution containing rabbit-anti vesicular acetylcholine transporter (VAChT, Nr. 139103, 1∶1000, Synaptic Systems, Goettingen, Germany) in PBS containing 2% normal goat serum and 0.2% Triton X-100. The next day the sections were processed for immunoperoxidase labelling. To do so, all sections were incubated for 30 min at room temperature in biotinylated secondoary antibodies (diluted 1∶300 in Tris-saline containing 2% normal goat serum) and processed for avidin-peroxidase staining (Vectastain Elite Kits, Vector Laboratories, Burlingame, CA). Up to 5 sections were kept in one well and stained for 5 to 10 minutes. After three washes in Tris-saline, sections were mounted on gelatinized glass slides and air-dried overnight.

### Quantification of immunohistochemical staining

The immunoperoxidase-stained slides were scanned with a slide-scanning microscope (Mirax Midi Slide Scanner; Zeiss) in bright-field mode at the level of the corpus callosum (Bregma 1.70 to 1.60). Images were acquired with a digital camera (1288×1040 pixels, with a pixel size of 0.23 um; AxioCam monochrome charge-coupled display; Zeiss) with a 20× objective (NA 0.8) using the software Pannoramic Viewer (version 1.14.25.1; 3D Histech Ltd, Budapest, Hungary). For quantitative analysis the ImageJ software was applied (Version 1.45s; NIH, USA) to export 6 randomly selected high-magnification JPG images, 3 in each hemisphere of the primary somatosensory cortex in layer II and III. All analyses were performed blinded related to age, condition and image. Each of the selected images was then converted into an 8-bit image. As done routinely, to correct for staining intensity each image was treated individually and a threshold was determined manually [39]. For further analyses the VAChT stained area of each image was expressed as percentage of total area.

## Results

### Sleep slow wave activity in juvenile rats shows an inverted U-shape trajectory as found in humans

**Figure 1:**
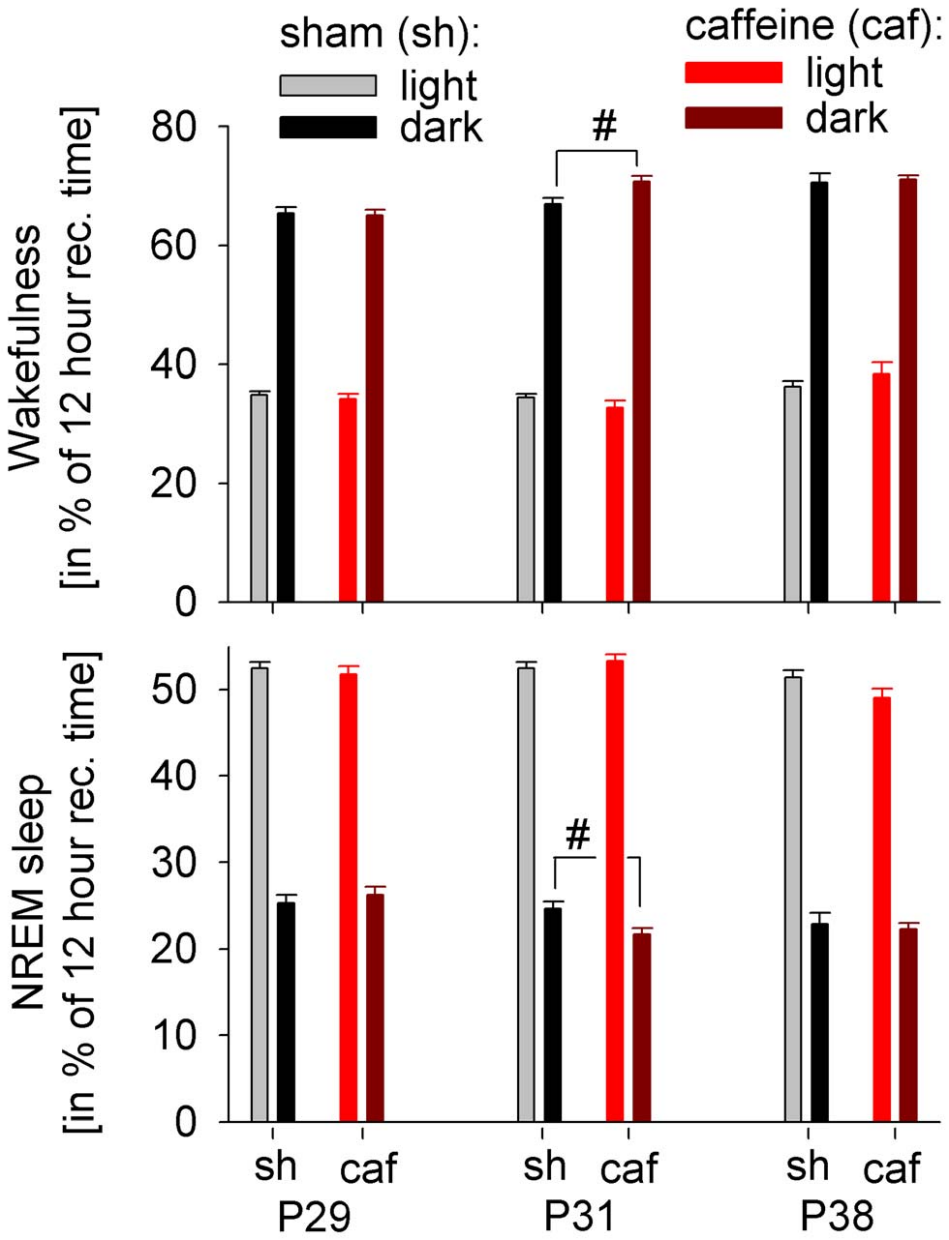
Vigilance states across age. Wakefulness and non rapid eye movement (NREM) sleep in sham (sh, n = 15) and caffeine (caf, n = 11) treated animals, expressed as a percentage of 12 hours recording time (rec. time) for the light and dark period before (P29), during (P31) and after (P38) caffeine treatment. A two-way repeated measures ANOVA with factor age (P29, P31 and P38) and condition (caffeine and sham) performed for NREM sleep and wakefulness during the light period was significant for age. The same analyses for NREM sleep and wakefulness during the dark period revealed an effect of age and an interaction between condition and age (all, p<0.05). The group comparison during caffeine application (P31) showed increased wakefulness and decreased NREM sleep during the dark period, respectively (#p<0.05, unpaired Student's t-test).

**Figure 2:**
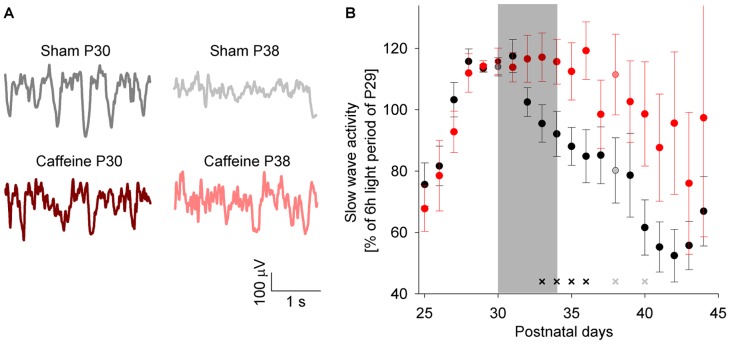
SWA trajectory across age in sham and caffeine treated animals. (**A**) Sample ECoG traces of a sham and caffeine treated animal on P30 and P38, respectively. (**B**) Trajectory of sleep slow wave activity (ECoG power between 1 and 4 Hz, averaged over the first 3 hours after light onset) between postnatal day 25 (P25) and P45 for sham (n = 17) and caffeine (n = 11) treated animals. The grey shaded background illustrates the period of caffeine administration. A two-way repeated measures ANOVA with factor age (P25–P45) and condition (caffeine and sham) was significant for age and condition (p<0.05). Crosses indicate increased SWA in caffeine compared to sham treated animals (black, p<0.05, gray, p<0.08), unpaired Student's t-test).

**Figure 3:**
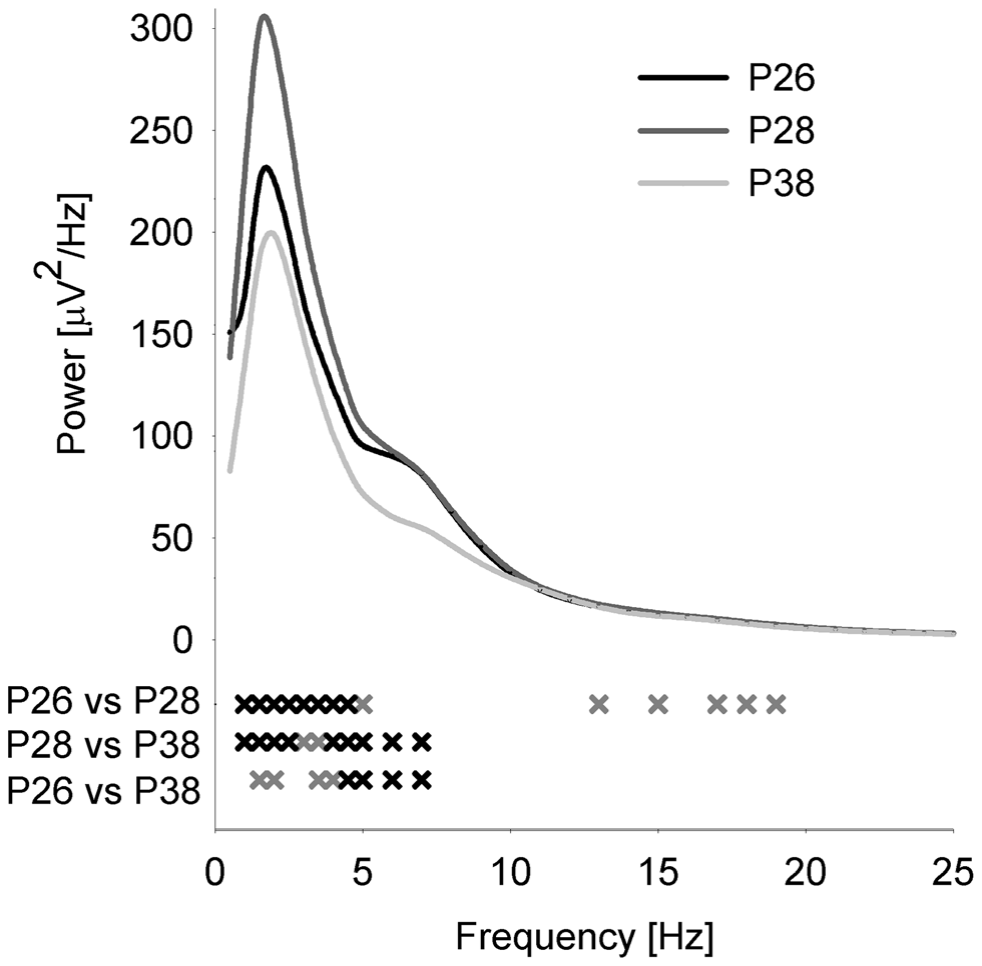
EEG NREM sleep spectrum across age. Average NREM sleep EEG power between 0.5 and 25 Hz across 24 hours for sham treated animals is shown for P26 (n = 17), P28 (n = 17) and P38 (n = 15). Crosses indicate power differences across age (grey, p<0.05, black, p<0.01), paired Student's t-test).

For the assessment of age-dependent changes in sleep, we longitudinally recorded electrocortical recordings (ECoG) in the Sprague Dawley rat after weaning, starting on postnatal day 25 (P25) for 20 consecutive days. First, we examined age-dependent changes of the vigilance states for selected days. We found an increase in wakefulness with increasing age during the light and the dark period, which was paralleled by a decrease in NREM sleep (Fig. 1). Next, we quantified age-related changes in the ECoG by performing spectral analysis. We focussed on sleep slow wave activity (SWA, ECoG power between 1 and 4 Hz during NREM sleep, averaged across the first 3 hours after light onset), as it was shown to exhibit prominent age-dependent changes in humans. SWA in the rat increased progressively during early development and reached a plateau during puberty (P30), before declining significantly during subsequent days (Fig. 2b). A comparison of the ECoG spectrum between 0.5 and 25 Hz showed that these age-dependent changes were most prominent in the SWA frequency range (Fig. 3).

**Figure 4:**
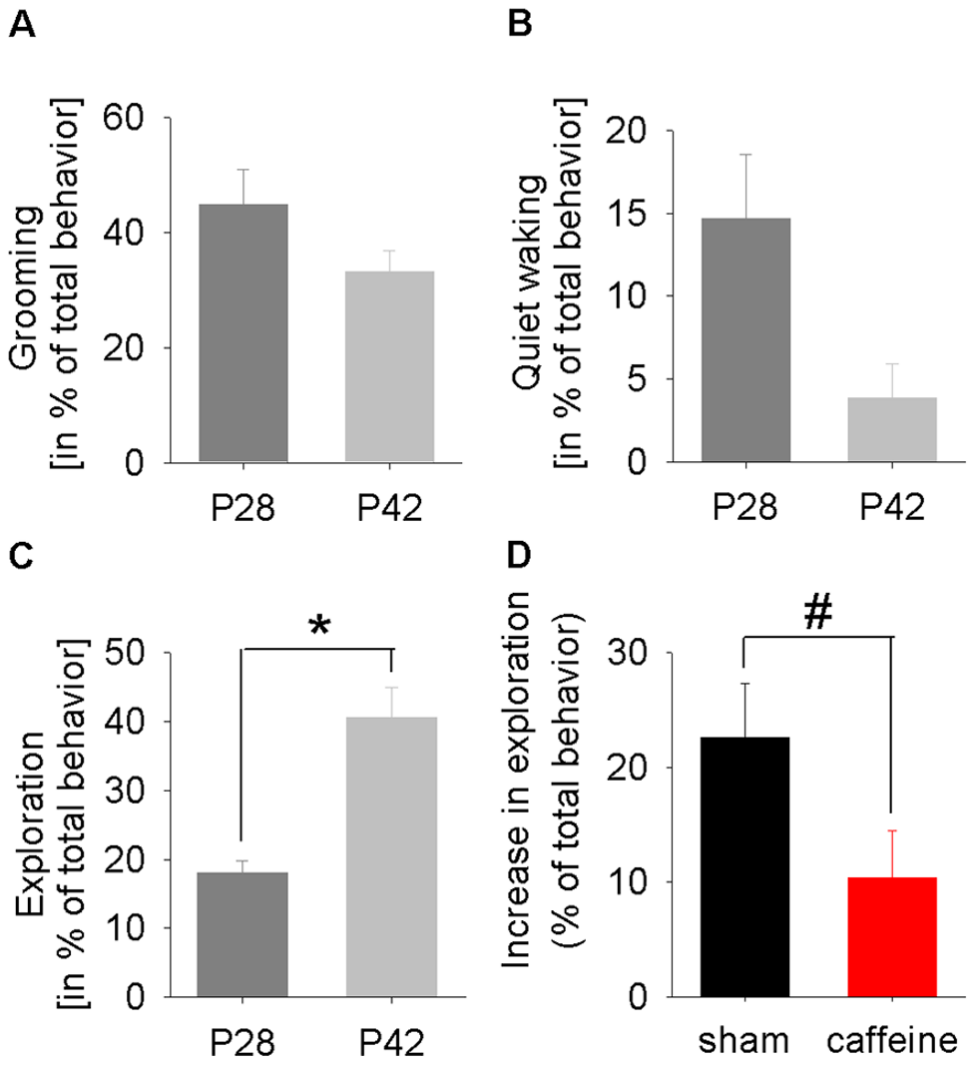
Behavioral changes across age. (**A, B, C**) The amount of grooming, quiet waking and exploration expressed as a percentage of total behavior are shown for P28 and P42. Significant changes across age are illustrated by an asterisk (p<0.05, paired Student's t-test). (**D**) The increase in object exploration time from P28 to P42 was reduced in caffeine (n = 9) compared to sham (n = 8) treated animals (#p<0.05, Mann-Whitney U-test).

The changes in SWA were paralleled by behavioral changes assessed in a repeated free exploration task (P28 and P42), during which more mature animals showed increased explorative behavior of a novel object (Fig. 4c). The other behavioral parameters (grooming, quiet waking see Fig 4a, b and general exploring, data not shown) did not change across age.

**Figure 5:**
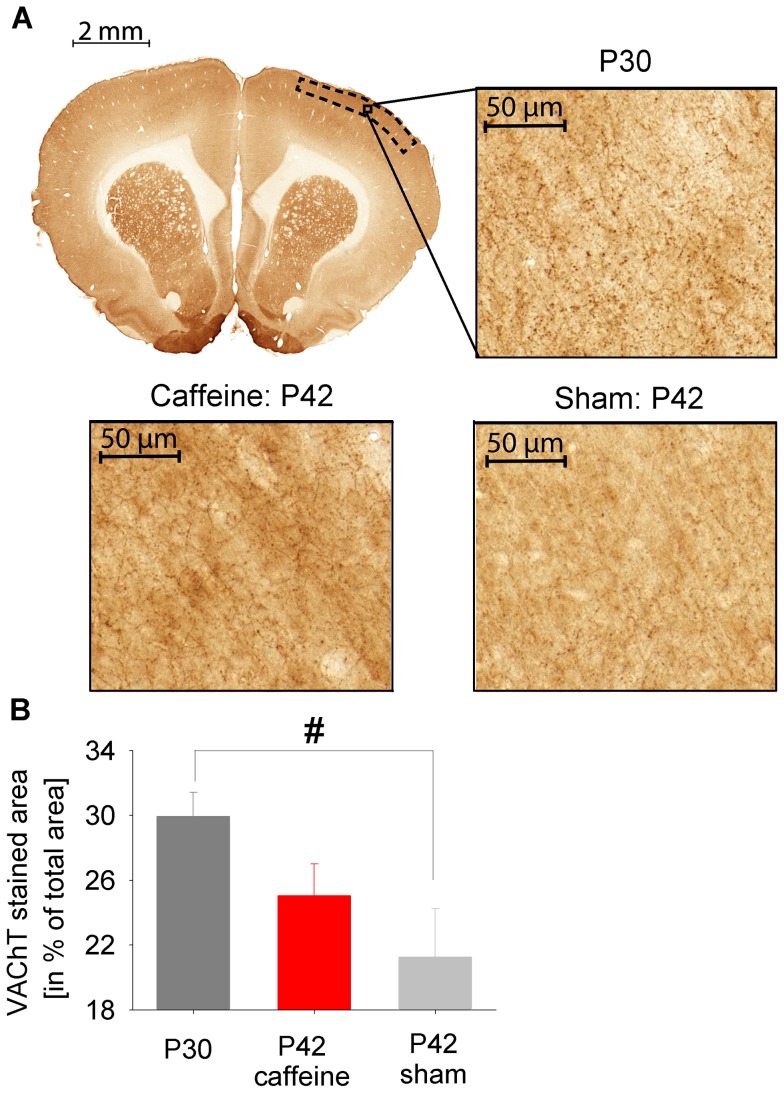
Structural changes across age. (**A**) Representative example image of a coronal section stained for vesicular acetylcholine transporter protein (VAChT), a specific marker for cholinergic presynaptic terminals. The dotted black box indicates the location of the randomly selected images for further analyses. One example, indicated by the solid black box is enlarged for postnatal day 30 (P30, right image). Below representative images for P42 after either sham or caffeine treatment are shown. (**B**) Reduction of the VAChT stained area, assumed to reflect cholinergic presynaptic terminals from P30 to P42 (n = 6 per group, *p<0.05, Mann-Whitney U-test). Caffeine treated animals show a diminished reduction of presynaptic cholinergic terminals at P42.

Given the supposedly close relationship between SWA and cortical plasticity, we assessed structural changes in coronal sections stained for VAChT, a marker for cholinergic presynaptic terminals before and after the critical period when SWA decreased (P30 and P42). Representative sections are provided in Figure 5a. A quantification showed a significant reduction of the VAChT stained area expressed as percentage of total area at older age (Fig. 5b).

To manipulate sleep wake regulation during the critical period when SWA started to decline on P30 we administered caffeine via the drinking water for 5 consecutive days.

### Caffeine exerts short-term stimulating effects

**Figure 6:**
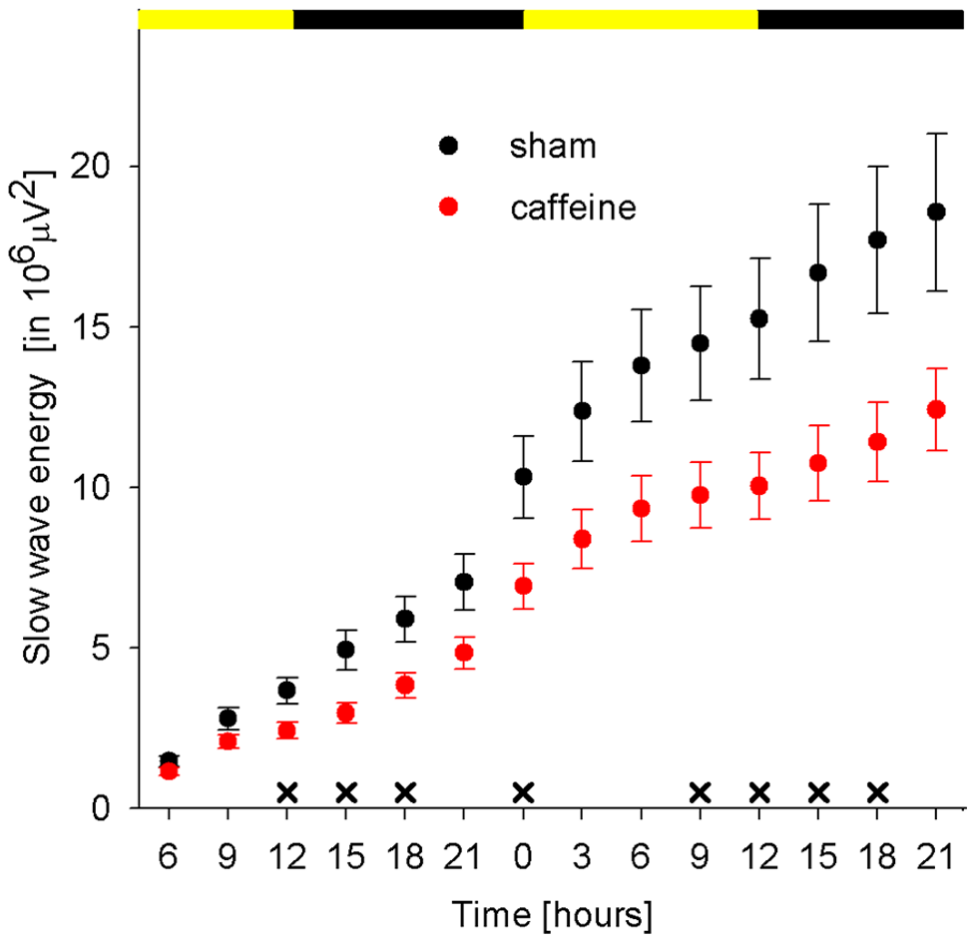
Caffeine reduces the build up of slow wave energy (SWE) during early caffeine treatment. Filled circles represent accumulated SWE (see Materials and Methods for details) in 3 hour intervals during the period when caffeine was initiated on P30 until the end of the second day of treatment (P31). The period of caffeine administration is illustrated by the grey shaded background. The white and black bars at the top of each graph indicate the 12 hour light and the 12 hour dark period, respectively. Crosses indicate reduced SWE in caffeine (n = 11) compared to sham (n = 17) treated animals (p<0.05, unpaired t-test). Error bars indicate SEM.

Moderate caffeine intake (16.0±1.4 mg/kg per day, see Materials and Methods for details) resulted in an expected increase in wakefulness and a reduction of NREM sleep during the dark period on the first day after the start of caffeine treatment (Fig. 1). No significant changes in vigilance states were observed in subsequent days (data not shown). Moreover, caffeine treatment led to a reduced build-up of slow wave energy (SWE), a measurement quantifying the accumulation of SWA across time and therefore controlling for the amount of wakefulness, during the initial two days of caffeine treatment (Fig. 6).

### Long-term effects of caffeine

After the end of caffeine treatment no changes in the amount of wakefulness and NREM sleep between the two groups were found (P35, data not shown; P38, see Fig. 1). However, we found significant changes in the trajectory of SWA in caffeine treated animals beyond the treatment period. Caffeine treated animals exhibited higher SWA, averaged over the first 3 hours after light onset, between P33 and P36. Trend level differences were still observed 5 days after caffeine treatment on P40 (Fig. 2b). The higher level of SWA in caffeine treated animals was observed across the entire 24 hours 1 day after caffeine treatment on P35 (data not shown). Moreover, while an age-dependent increase in novel object exploration was found in both groups (for sham, see Fig. 4c), the increase in exploration time was reduced in caffeine treated animals compared to untreated animals (Fig. 4d). The immunohistochemical analysis under sham maturation showed a decrease in the VAChT stained area across age, a marker for cholinergic presynaptic terminals. After caffeine treatment this area was diminished (Fig. 5b).

## Discussion

This study used a combination of electrocortical (ECoG) recordings, behavioral and structural readouts to track maturational changes in the juvenile rat and their manipulation by caffeine. Our findings show that sleep slow wave activity (SWA) in the rat follows a similar inverted U-shaped trajectory as already known for humans [18], [40], [41], [42]. Furthermore, mild caffeine treatment during the critical period when sleep SWA normally declines (between postnatal 30 (P30)-P34) affected sleep and resulted in alterations in maturational parameters. First, the SWA decline was delayed after caffeine treatment. Second, caffeine treatment reduced the normal increase in exploratory behavior. And last, the decrease of a marker for cholinergic presynaptic terminals across age was diminished in caffeine treated animals.

In humans it is well known that SWA during NREM sleep shows prominent age-dependent changes during the first two decades of life [20], [41], [43], [44], [45]. It was repeatedly shown that initially SWA increases, peaks shortly before puberty, and decreases during adolescence. Our results show a similar inverted U-shaped trajectory of SWA in the juvenile rat compared to humans. Moreover, peak SWA in humans is reached before puberty. Correspondingly, SWA in the rat peaks on P30, a period which was related to the beginning of puberty [46]. Therefore, SWA in both species follows a similar trajectory during a comparable developmental period suggesting the rat to be a good model for further investigations. The age-dependent changes, though most pronounce, were not entirely restricted to the SWA frequency band but extended in the theta frequency range (6–7 Hz). This observation is in line with other studies reporting age-dependent changes which are not only restricted to the SWA frequency range [20], [44]. Moreover, sleep deprivation studies show that the prominent increased power in the SWA band extends into the theta frequency range [47].

Due to the similar trajectory of synapse density and SWA, Campbell and colleagues suggested that the decrease of synapse density during adolescence is reflected in the decrease of SWA during this developmental period [40]. A mechanistic explanation for this close relationship is based on the observation that synchronization of cortical activity is a key factor determining the level of SWA [23]. Thus, increased synaptic density would enable faster synchronization of network activity, resulting in more SWA. Our microstructural data of the VAChT stained area provides a measure for presynaptic cholinergic terminals in the cortex [48]. Moreover, it was shown that VAChT overexpression leads to increased acetylcholine (Ach) release and to an increased amplitude and frequency of miniature excitatory postsynaptic currents [49]. These results suggest a close relationship between VAChT and cholinergic signalling. Thus, the reduction of VAChT stained area seems to reflect a reduction in cholinergic signalling which parallels the reduction of SWA from P30 to P42. Together with evidence that the entire cerebral cortex matures as an integrated network [29], our results support the proposed mechanistic relationship between synaptic density and SWA in the rat. However, our interpretation is based on the assumption that VAChT also during cortical development provides a good measure for functional cholinergic synapses. This assumption is supported by a study in rats showing the highest Ach transmitter release, the best marker for functional synapses, on P30 [37]. This goes along with the proposed function of Ach during postnatal development providing a favourable environment for neuronal plasticity processes [33], [34], [35]. Several studies provide additional evidence for structural changes to occur during the inverted U-shape trajectory of SWA. For example, an *in vitro* study in rats showed a similar inverted U-shaped trajectory of synapses per neuron peaking also around P30 [50]. Moreover, markers of postsynaptic density (PSD) show a similar developmental trajectory. For example, it was shown that postsynaptic protein 95 (PSD-95), a marker protein of postsynaptic density [51] involved in maturation of excitatory synapses [52], shows age-dependent changes. PSD-95 was massively increased at the beginning of the 4th postnatal week compared to postnatal week 1 and postnatal week 9 [53]. Interestingly, PSD was found to be regulated in an activity-dependent manner [54].

We did not find evidence for a direct link between sleep variables, behavioral and structural changes. This is or might be due to the following reasons: Correlations between structural findings and sleep ECoG changes are not possible since the animals needed to be killed at the selected time points. In addition, the number of animals for which we have the combined measures (behavior and ECoG) is rather low making it difficult to find correlative evidence. A limitation, which has to be considered when looking at the trajectory of sleep SWA, is that data collection started immediately after surgical termination which did not allow the animal to undergo a recovery period. However, for our main findings, i.e. the decrease of SWA during the post-pubertal period (e.g after P30) and the diminished reduction of SWA after caffeine application, the animals had at least 5 days of recovery after surgery. Even tough we did not expect any post surgical effects on our main findings we cannot exclude any impact of the surgery on the prior increasing portion of the SWA trajectory (between P26 and P30). A repeated measure ANOVA with day of surgery as a between-subject factor (P23, P24 and P25) and SWA trajectory (age) as a within-factor showed a significant effect of age. However, no interaction between age and day of surgery was found, providing good evidence that the recovery period did not affect the recording also during the early days (between P26 and P30). Moreover, we have conducted a control experiment during which the same surgical procedure followed by ECoG recordings was applied in animals at older age. The same analyses did not show an inverted U-shaped trajectory as observed in juvenile rats (data not shown).

Caffeine affected sleep in juvenile rats. In line with the literature, we found a similar short-term effect of caffeine on vigilance states. Initial caffeine treatment led to increased wakefulness and decreased NREM sleep [6]. A caffeine induced increase in wakefulness was shown to be associated with an increase in SWA during subsequent sleep periods [6]. In contrast, our rather small increase in wakefulness during the dark period did not result in more SWA at the beginning of the next light period (Fig. 2, P32). This discrepancy might be due to different aims of the caffeine administration. Schwierin and colleagues aimed to perform a pharmacological sleep deprivation for approximately 4 hours by injecting caffeine intraperitoneally at light onset. However our rationale was not to increase wakefulness and we therefore administered caffeine via the drinking water during a time window the animals are naturally awake. One established way to correct for even small changes in the amount of wakefulness, e.g. like during our early caffeine treatment, is to calculate SWE. We found that SWE was reduced in caffeine treated animals. This suggests that caffeine reduces the build up of sleep pressure during waking as already proposed previously [6], [7].

One limitation of our approach was that caffeine administration was performed via the drinking water in order to not induce any stress, which was shown to directly influence SWA [55], one of our main parameters. The administration of caffeine via the drinking water provided a measure of overall daily caffeine consumption but did not allow further specification about the circadian time (between 6 pm–2 am) when caffeine was consumed.

Caffeine also led to long-term effects on the SWA trajectory. More precisely, we observed a delayed reduction of sleep SWA after caffeine application. Interestingly, we found similar results for our behavioral and structural marker of maturation. More specifically, 7 days after the end of caffeine administration we found behavioral and structural changes: 1) Caffeine treatment led to a less mature behavior as assessed by object exploration, which increased as a function of age. 2) VAChT stained area, presumably reflecting mainly cholinergic presynaptic terminals, significantly decreased across age, while caffeine treatment led to a diminished reduction. In conclusion, these results propose that caffeine has long lasting effects on maturation and the SWA trajectory closely parallels these changes. These parallel trajectories strengthen the observation that sleep SWA might be used as an electrophysiological marker of cortical maturation [56], [57]. That caffeine is able to interfere with the trajectory of brain morphological changes during development has been shown previously. For example, caffeine was shown to have a long lasting effects on morphological parameters such as dendritic length in the neonatal rat in the prefrontal cortex [58] as well as in the middle age rat in the hippocampus preventing cognitive decline [59]. Knockout models may help to explore the underlying mechanism of a potential role of adenosine signalling during cortical maturation. Several studies explore the effects of adenosine A_1_, A_2A_, A_2B_ or A_3_ receptors in single knockout mice [60], [61]. However, our applied caffeine dose mainly affects A_1_ and A_2A_ receptors and no study investigates how cortical maturation is affected during a similar developmental period in A_1_, A_2A_ receptors double knockout mice. Thus, a potential role of adenosine via adenosine receptors during cortical development has not been explored yet. Moreover, whether or not such caffeine-induced changes in morphology are due to alterations in sleep regulatory processes has not been investigated. Our data indicate that this might be the case. This interpretation is supported by the synaptic homeostasis hypothesis [62] which proposes a key role of sleep slow waves in synaptic plasticity. Thus, altering sleep wake regulation by caffeine, reflected in changes of SWA, may affect synaptic plasticity. Nevertheless, there are alternative explanations of how caffeine may interfere with synaptic plasticity. For example, caffeine reduces the number of microglia [63] which seem to be important for synaptic pruning [64]. Or, caffeine blocks the activation of Cofilin [65], an actin binding protein, which is thought to be important for synaptic plasticity [66].

No matter what mechanism applies our study shows that caffeine interferes with cortical maturation during a critical developmental period. This might also be of clinical importance since the critical period of synapse elimination during adolescence is associated with an increasing incidence of psychiatric and mood disorders, such as schizophrenia, anxiety, substance abuse and personality disorders [67]. Thus, it will be important for future studies to investigate the effects of chronic caffeine application during critical periods of development in animal models for psychiatric and mood disorders.
